# Oxidative and Inflammatory Damage by Environmental Polyethylene Microplastics in Caco‐2 Cells Is Prevented by Polyphenol‐Rich Limoncella Apple Extract

**DOI:** 10.1155/omcl/3136395

**Published:** 2026-02-24

**Authors:** Stefania Lama, Massimo Venditti, Alessandra Biasi, Carmen Lenti, Hana Najahi, Mohamed Banni, Gian Carlo Tenore, Ettore Novellino, Paola Stiuso

**Affiliations:** ^1^ SSD Cryopreservation and B.a.S.C.O., Oncohematology Department AORN Santobono-Pausillipon, Naples, Italy; ^2^ Department of Experimental Medicine, University of Campania “Luigi Vanvitelli”, Naples, 80138, Italy, unina2.it; ^3^ Department of Precision Medicine, University of Campania “Luigi Vanvitelli”, Naples, 80138, Italy, unina2.it; ^4^ Higher Institute of Biotechnology, University of Monastir, Monastir, 5000, Tunisia, um.rnu.tn; ^5^ Department of Pharmacy, University of Naples Federico II, Via Domenico Montesano 49, Naples, 80131, Italy, unina.it; ^6^ Faculty of Medicine and Surgery, Catholic University of the Sacred Heart, Rome, 00168, Italy, unicatt.it; ^7^ Unit of Dietetics and Sports Medicine, University of Campania “Luigi Vanvitelli”, Naples, 80138, Italy, unina2.it

**Keywords:** apple polyphenol extract, differentiated Caco-2 cells, environmental polyethylene microplastics, epithelial-mesenchymal transition, nutraceuticals, oxidative stress, polyphenols, tight junctions

## Abstract

**Background:**

Humans are constantly exposed to environmental microplastic (MP) particles, which can be absorbed through the gut and exert adverse health effects. This study aimed to investigate the harmful effects of environmental polyethylene MPs (PE, 2.6 μm) on differentiated Caco‐2 (D‐Caco‐2) intestinal epithelial cells and to assess the protective potential of *Limoncella* apple polyphenol extract (LAPE).

**Methods:**

D‐Caco‐2 cells were exposed to PE, LAPE, or their combination. Cell viability and lipid peroxidation were evaluated using MTT and TBARS assays, respectively. The organization of F‐actin and alkaline phosphatase proteins was evaluated by immunofluorescence, whereas occludin and NF‐κB were evaluated by Western blot analysis.

**Results:**

PE reduced D‐Caco‐2 viability and impaired cell differentiation by increasing lipid peroxidation. In addition, PE destructured F‐actin organization and altered the expression of occludin, a tight‐junction protein.

**Conclusions:**

Our findings show that PE increases oxidative stress, triggering epithelial–mesenchymal transition and dedifferentiation in Caco‐2 cells. Interestingly, LAPE, owing to its antioxidant and anti‐inflammatory properties, counteracted the harmful effects of PE, suggesting its potential as a nutraceutical strategy to prevent MP‐induced damage in the gastrointestinal (GI) tract.

## 1. Introduction

Among the most pervasive environmental contaminants of the modern era, plastics and microplastics (MPs) have emerged as critical pollutants due to their persistence and widespread distribution. These particles are now ubiquitous in terrestrial and aquatic ecosystems and have been detected across multiple trophic levels, ultimately reaching human tissues through dietary exposure. An estimated 20 million metric tons of plastic are produced every year, mostly deriving from the food packaging industry [1].

MPs are defined as solid plastic fragments smaller than 5 mm, either intentionally manufactured or formed progressively through the breakdown of larger plastic debris [2]. The most used varieties of MPs are polyethylene, polypropylene, poly (ethylene terephthalate) (PET), and polystyrene (PS) [3].

Animal studies have demonstrated that, following absorption, MPs can translocate to multiple organs, including the liver, kidneys, spleen, lungs, reproductive tissues, and even the central nervous system, raising concerns about their systemic toxicity [4].

A major route of exposure to MPs is ingestion, but their potential effects in the gastrointestinal (GI) tract remain unclear. However, recent evidence has demonstrated that GI exposure to MPs induces an imbalance in oxidative and inflammatory homeostasis, along with increased epithelial permeability in the murine gut [5]. Yan et al. [6] have shown that MPs concentrations in feces were significantly higher in patients with inflammatory bowel disease compared to healthy individuals.

Compromise of the intestinal epithelial barrier is recognized as a key contributor to the pathogenesis of various chronic inflammatory conditions affecting the GI tract. Intestinal tight‐junction proteins play an essential role in maintaining epithelial integrity and regulating permeability, thus preventing the translocation of toxins and pathogens from the lumen into the bloodstream [7].

During oxidative stress, excessive free radical production alters the structure of tight‐junction proteins, leading to immune activation. In a vicious cycle, prolonged exposure to inflammatory stimuli results in the loss of enterocyte polarity and differentiation, ultimately progressing to epithelial–mesenchymal transition (EMT).

Polyphenols encompass a diverse group of bioactive compounds with well‐documented antioxidant and anti‐inflammatory properties, which contribute significantly to the preservation of intestinal barrier integrity. Among them, the polyphenols curcumin, quercetin, epigallocatechin‐3‐gallate, and resveratrol have been reported to inhibit Wnt/*β*‐catenin signaling pathways [8]. In the intestinal epithelium, this pathway is crucial for stem cell proliferation and differentiation within the crypts, as well as for EMT and the aberrant expansion of cancer stem cells (CSCs).

Recently, a specific polyphenol extract from the “Limoncella Apple” (LAPE) has been shown to significantly reduce serum lipid peroxidation and liver injury in DNBS‐induced colitis, as well as to suppress nuclear factor kappa B (NF‐κB) activation [9].

In this paper, we evaluated the toxicity of environmental polyethylene MPs (PE) in differentiated Caco‐2 (D‐Caco‐2) cells, a good model of human intestinal enterocytes. Furthermore, we investigated the protective role of LAPE on cell viability, lipid peroxidation, differentiation, and EMT in PE‐treated D‐Caco‐2 cells. D‐Caco‐2 cells were selected as an in vitro model because they spontaneously differentiate into enterocyte‐like monolayers expressing brush‐border enzymes, transporters, and tight‐junction proteins. However, we acknowledge that Caco‐2 cells originate from a colorectal adenocarcinoma and exhibit altered metabolic and redox characteristics compared to nontransformed intestinal epithelial cells. These limitations are discussed in detail in the Discussion section, and future studies will extend these findings to nontransformed intestinal models.

## 2. Materials and Methods

### 2.1. Chemicals

Cell culture plastics were obtained from Becton Dickinson (Lincoln Park, NJ, USA). Fetal bovine serum (FBS), phosphate‐buffered saline (PBS), L‐glutamine, trypsin, and antibiotics were purchased from Gibco (Life Technologies, Carlsbad, CA, USA). Minimum Essential Medium Eagle Alpha Modification (α‐MEM) was obtained from Gibco, Thermo Fisher Scientific, Inc. (Waltham, MA, USA). All other reagents used were of analytical grade.

### 2.2. Preparation of Environmental PE and LAPE

PE solutions were prepared based on the protocols described by Zuccarello et al. [10], with minor modifications to fit our experimental conditions. Stock solutions were autoclaved for 40 min prior to use. PE (2.6 μm in diameter) was added to D‐Caco‐2 at concentrations of 2.5 and 5 μg/mL for 24, 48, and 72 h. This particle size was selected because it is commonly detected in bottled water [9]. The in vitro GI digestion of *Limoncella* apple polyphenol extract (LAPE) used in the present study originates from the same batch that was previously extracted, fully characterized, and described in detail in our earlier publication Lama et al. [9]. Its polyphenol content was analyzed using HPLC/DAD (Jasco Extrema LC‐4000 system, Jasco Inc., Easton, MD, USA). Chromatographic separation was achieved with a Kinetex C18 column (250 mm × 4.6 mm, 5 μm; Phenomenex, Torrance, CA, USA). Detection wavelengths were set at 280 nm and 320 nm to monitor flavan‐3‐ols and hydroxycinnamic acids, respectively. The major constituents of this batch were catechin, epicatechin, and chlorogenic acid. In all experiments, LAPE was used at a final concentration of 80 μg/mL.

### 2.3. Cell Viability

Caco‐2 cells (21st passage; ATCC, Rockville, MD, USA) were cultured at 37°C in high‐glucose MEM supplemented with 1% (v/v) nonessential amino acids, 10% (v/v) heat‐inactivated FBS (Flow, McLean, VA), 100 U/mL penicillin, 100 μg/mL streptomycin, 1% L‐glutamine, and 1% sodium pyruvate. To generate D‐Caco‐2, cells were seeded at appropriate densities and exposed to PE at 2.5 or 5 μg/mL for 24, 48, or 72 h. Cell viability was assessed via the MTT assay. A solution of 3‐(4,5‐dimethylthiazol‐2‐yl)‐2,5‐diphenyltetrazolium bromide (5 mg/mL in PBS) was added to the cells to evaluate mitochondrial metabolic activity. Absorbance was measured at 570 nm using a Bio‐Rad 550 microplate reader (Bio‐Rad Laboratories, Milan, Italy). Results were expressed as a percentage relative to untreated controls. The effects of PE, LAPE, and their combination were also tested on DH‐Caco‐2 cells (D‐Caco‐2 cells pretreated with 50 μM H_2_O_2_ for 30 min). All experiments were performed in triplicate.

### 2.4. Permeability Assay

To assess intestinal barrier integrity, D‐Caco‐2 cells were seeded on transwell inserts (8 μm pore size) at a density of 2.0 × 10^5^ cells per well and cultured for 21 days to allow differentiation. Cells were then treated with PE, LAPE, or their combination for 72 h. After treatment, a mannitol–lactulose solution (0.05 mmol/L mannitol, 0.25 mol/L lactulose) was added to the apical chamber. After 3 h, the basolateral medium was collected and analyzed for mannitol and lactulose content using liquid chromatography‐mass spectrometry (LC‐MS) [7].

### 2.5. Western Blot Analysis

Total protein was extracted using RIPA lysis buffer (#TCL131; Hi Media Laboratories GmbH, Einhausen, Germany) supplemented with protease inhibitors (#39102; SERVA Electrophoresis GmbH, Heidelberg, Germany), followed by centrifugation at 14,000× *g* for 20 min. Protein concentrations were determined using the Lowry method. Equal amounts of protein (20 μg) were resolved by SDS‐PAGE (9%–15% polyacrylamide; #A4983; AppliChem GmbH, Darmstadt, Germany) and transferred onto PVDF membranes. Membranes were blocked for 1 h at room temperature in a buffer containing 20 mM Tris (pH 7.5), 500 mM NaCl, 5% nonfat dry milk, and 0.2% Tween‐20, then incubated overnight at 4°C with the following primary antibodies: occludin (1 : 1000; #33‐1500, Thermo Fisher Scientific), NF‐κB (1 : 1000; #E‐AB‐65807, Elabscience), and GAPDH (1 : 5000; #97166, Cell Signaling Technology). After washing, membranes were incubated with HRP‐conjugated secondary antibodies (goat anti‐rabbit and goat anti‐mouse, 1 : 5000; #PI‐1000 and #RSA1122, respectively) and developed using West Pico Supersignal‐ECL detection reagents (Amersham, Piscataway, NJ, USA).

### 2.6. Alkaline Phosphatase Activity

ALP activity, an indicator of enterocyte differentiation, was determined using a commercial ALP assay kit (Sigma Diagnostics, Number 245). Cells were lysed with 0.25% sodium deoxycholate, and ALP activity was normalized to total protein content and expressed as units per milligram of protein.

### 2.7. Immunofluorescence Labeling

D‐Caco‐2 monolayers treated with PE, LAPE, or their combination for 72 h were washed with PBS and fixed/permeabilized in 100% ethanol at −20°C for 20 min. Nonspecific binding was blocked using 5% BSA in PBS with 0.1% Tween‐20 for 1 h at room temperature. Monolayers were incubated overnight at 4°C with primary antibodies against β‐catenin and ALP (1 : 400; #E‐AB‐81527, Elabscience). After washing, cells were incubated with secondary antibodies: goat anti‐rabbit Alexa Fluor 488 (1 : 500; #A3273, Thermo Fisher Scientific) and goat anti‐mouse CF 568 (1 : 250; #SAB4600082, Sigma–Aldrich). F‐actin was visualized using fluorescein–phalloidin (0.3 μM; #51927, Sigma–Aldrich) for 30 min at 37°C. Nuclei were counterstained with DAPI, and slides were mounted using Mowiol. Images were captured using a Zeiss LSM 510 confocal microscope with a Plan–Apochromat 63 × /1.4 NA oil immersion objective.

### 2.8. TBARS Assay

Lipid peroxidation was assessed in D‐Caco‐2 and DH‐Caco‐2 cell homogenates treated for 72 h with PE (2.5 μg/mL), LAPE (80 μg/mL), or their combination. Homogenates were incubated with 0.5 mL of 20% acetic acid (pH 3.5) and 0.5 mL of 0.78% thiobarbituric acid, then heated at 95°C for 45 min. Samples were centrifuged at 4000 × *g* for 5 min, and TBARS content was measured spectrophotometrically at 532 nm, as described by Tenore et al. [7]. Results were expressed as μM TBARS per μg of protein. Each value represents the average of triplicate samples from two independent experiments. TBARS was selected as a key endpoint because lipid peroxidation is a primary mechanism of oxidative injury induced by MPs. Together with LDH release, these complementary markers allow a comprehensive evaluation of redox imbalance, membrane disruption, and oxidative cytotoxicity.

### 2.9. Statistical Analysis

All results are expressed as mean ± SEM from at least three independent experiments. Statistical comparisons were performed using one‐way ANOVA. Differences were considered statistically significant at *p* < 0.05.

## 3. Results

In this study, we investigated the harmful effects of environmental PE MPs particles (PE, 2.6 μm) on the Caco‐2 cell line. All treatments were performed on D‐Caco2, as this is a well‐established in vitro cellular model to reproduce the absorptive enterocytes of human intestinal epithelium. Additionally, we evaluated whether the antioxidant and anti‐inflammatory properties of a digested apple peel extract, “Limoncella” (LAPE), could mitigate the negative effects induced by PE. The composition of LAPE, analyzed by HPLC/DAD, revealed a high percentage of catechin and chlorogenic acid [11]. In all experiments, LAPE was used at a final concentration of 80 μg/mL, a dosage that does not exhibit cellular toxicity in D‐Caco‐2 cells (data not shown) and was selected based on prior in vivo efficacy studies in mice, where LAPE was active at 300 mg/kg. Considering typical oral bioavailability (~10%) and volume of distribution (~0.3 L/kg), this corresponds to an estimated plasma concentration of~100 μg/mL, supporting the physiological relevance of the chosen concentration.

### 3.1. LAPE Counteracts the Cytotoxic Effects of Environmental PE by Decreasing Lipid Peroxidation

To evaluate the harmful effect of PE on D‐Caco‐2 cells, a cytotoxicity assessment using the MTT assay was performed after 24, 48, and 72 h of treatment (Figure 1, panel A). We observed that 2.5 μg/mL of PE reduced D‐Caco‐2 cell viability by ~50% after 72 h compared to untreated control cells (CTR). Interestingly, as the concentration of PE increased, a reduction in the cytotoxic effect on D‐Caco‐2 cells was observed. This phenomenon might be explained by the aggregation capacity of PE in aqueous solution, which could lead to decreased cellular uptake.

Figure 1Effects of PE, LAPE, and PE/LAPE combination on D‐Caco‐2 cell viability. (a) Evaluation, by MTT assay, of D‐Caco‐2 cell viability treated with 2.5 and 5 μg/mL PE after 24, 48, and 72 h of incubation. (b) D‐Caco‐2 viability after 72 h of incubation with 2.5 μg/mL PE, 80 μg/mL LAPE alone or in combination (20 × 10^3^ cells per well in 96‐well plates).  ^∗^ *p* < 0.05,  ^∗∗^ *p* < 0.01.

**Figure:**
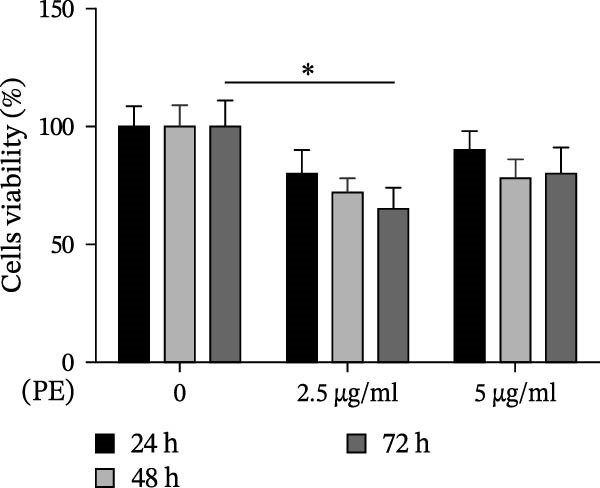
(a)

**Figure:**
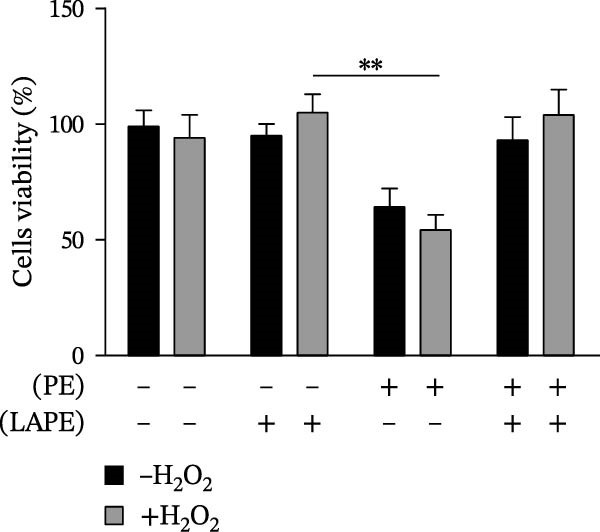
(b)

The addition of LAPE (80 μg/mL), either in combination with or following PE treatment (2.5 μg/mL), counteracted the cytotoxic effects on D‐Caco‐2 cells after 72 h (Figure 1, panel B).

Furthermore, we investigated whether oxidative stress could exacerbate the cytotoxic effects of PE on D‐Caco‐2 cells. Hydrogen peroxide‐exposed D‐Caco‐2 cells (HD‐Caco‐2) were treated with PE, and their viability was evaluated. PE‐treated HD‐Caco‐2 cells exhibited ~45% cytotoxicity (Figure 1, panel B) compared to HD‐Caco‐2 CTR. This effect was mitigated only when LAPE was added in combination with PE.

Based on these findings, all subsequent experiments were performed with the simultaneous addition of the PE/LAPE combination (2.5 μg/80 μg per mL) to D‐Caco‐2 cells for 72 h.

The effect of PE, LAPE, and their combination on lipid peroxidation was assessed using the thiobarbituric acid reactive substances (TBARS) assay (Figure 2 panel A). The results indicated that PE MPs increased lipid peroxidation (Figure 2A) in both D‐Caco‐2 and HD‐Caco‐2 cells compared to CTR (CTR vs. PE in D‐ and DH‐Caco‐2 cells *p* = 0.0056 and 0,011 respectively). This effect was significantly reversed by the PE/LAPE combination in both treated cell groups (PE vs. PE/LAPE *p* = 0.0001 in DH‐Caco‐2).

Figure 2(a) Effects of PE, LAPE, and PE/LAPE combination on lipid peroxidation of D‐ and DH‐Caco‐2 cells evaluated by TBARS assay. (b) Extracellular lactate dehydrogenase (LDH) assay on D‐Caco‐2 and DH‐Caco‐2 cells as a percentage of the total cellular LDH activity. The cells were incubated for 72 h with and without PE, LAPE, and PE/LAPE combination. Each point represents the mean ± SD of three experiments (*p* value < 0.05 compared to the negative control).  ^∗^ *p* < 0.05,  ^∗∗^ *p* < 0.01,  ^∗∗∗^ *p* < 0.001.

**Figure:**
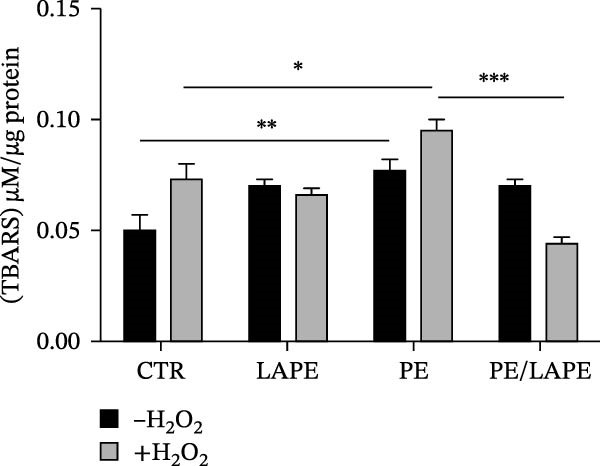
(a)

**Figure:**
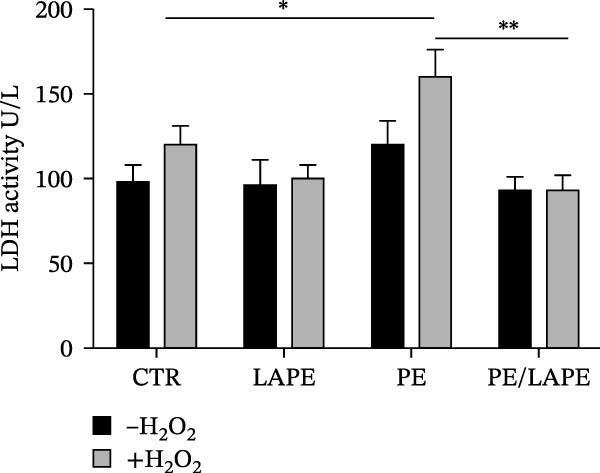
(b)

When H_2_O_2_ induces oxidative damage in Caco‐2 cells, the permeability of the cell membrane increases, promoting the release of intracellular LDH into the external environment. Therefore, LDH can be used to reflect the degree of oxidative damage in Caco‐2 cells. As shown in Figure 2B, the LDH content in the cell culture medium under normal culture conditions of both D‐Caco‐2 and DH‐Caco‐2 cells was 90–100 U/L and 120–130 U/L, respectively. We did not observe any variation in LDH content in either D‐Caco‐2 or DH‐Caco‐2 cells after LAPE treatment. When the D‐Caco‐2 and DH‐Caco‐2 cells were stimulated with PE, the LDH content in the medium was 120 U/L and 160 U/L, respectively. In PE‐treated DH‐Caco‐2 cells, the increase was about 33% compared to untreated DH‐Caco‐2 cells. The antioxidant effect of LAPE, both alone and in combination with PE, restored LDH values to levels comparable to those of D‐Caco‐2 cells. (PE vs. LAPE combination in DH‐Caco2 cells *p* = 0.0032).

### 3.2. PE‐Induced Hyperpermeability, Decrease Occludin and NF‐κB Expression in D‐Caco‐2 Cells

It is noteworthy that MPs have been reported to induce intestinal epithelial barrier injury by increasing oxidative stress [12]. We therefore explored whether the antioxidant properties of LAPE could restore intestinal barrier permeability.

The D‐Caco‐2 monolayers grown in the Transwell system were used to evaluate cellular permeability to ions and solutes. Intestinal permeability was assessed using the lactulose/mannitol (L/M) 5 : 1 molar ratio, a well‐established marker of intestinal permeability.

Treatment of D‐Caco‐2 cells with PE increased the L/M ratio in the basolateral solution by ~3‐fold, compared to CTR (Table 1). Interestingly, the PE/LAPE combination restored cell permeability, returning the L/M ratio to near‐baseline levels observed in CTR (cells without treatment).

**Table 1:** Effects of PE, LAPE, and PE/LAPE combination on D‐Caco‐2 cell permeability evaluated by lactulose/mannitol ratio.

Treatment	Basolateral compartment L/M ratio
CTR	0.35 ± 0.07
PE	1.1 ± 0.12
LAPE	0.47 ± 0.1
PE/LAPE	0.38 ± 0.09

*Note*: L/M ratio.

To further examine the intestinal epithelial barrier dysfunction induced by PE, the expression of occludin was examined by western blot analysis. As shown in Figure 3, PE‐treated D‐Caco‐2 cells significantly decreased occludin expression, which was reversed by the PE/LAPE combination compared to CTR cells. The anti‐inflammatory properties of polyphenol extracts are known. Here we analyzed, by western blot analysis, the changes in NF‐κB protein level in D‐Caco‐2 cells exposed to PE, LAPE, and PE/LAPE combination. Our data showed that NF‐κB decreases in both LAPE and PE/LAPE combination‐treated D‐Caco‐2 cells compared to CTR cells.

Figure 3(a) Western blot analysis of occludin, and NF‐κB in the PE, LAPE, and PE/LAPE combination‐treated D‐Caco‐2 cells. (b) Histograms showing the relative protein levels of occludin and NF‐κB normalized with GADPH. Values are expressed as means ± SEM from 3 different experiments.  ^∗^ *p* < 0.05,  ^∗∗^ *p* < 0.01,  ^∗∗∗^ *p* < 0.001.

**Figure:**
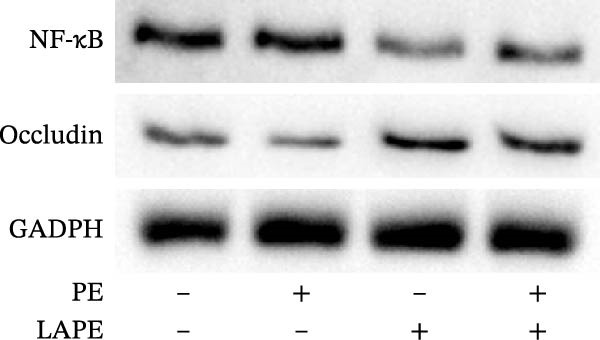
(a)

**Figure:**
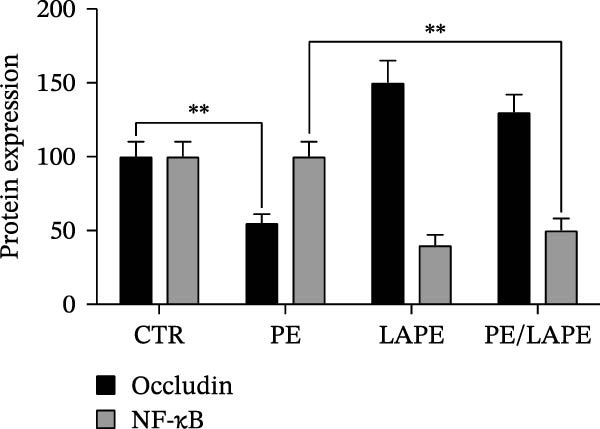
(b)

### 3.3. PE‐Induced Epithelial Mesenchymal Transition and Dedifferentiation of D‐Caco2 Cells

**Figure 4:**
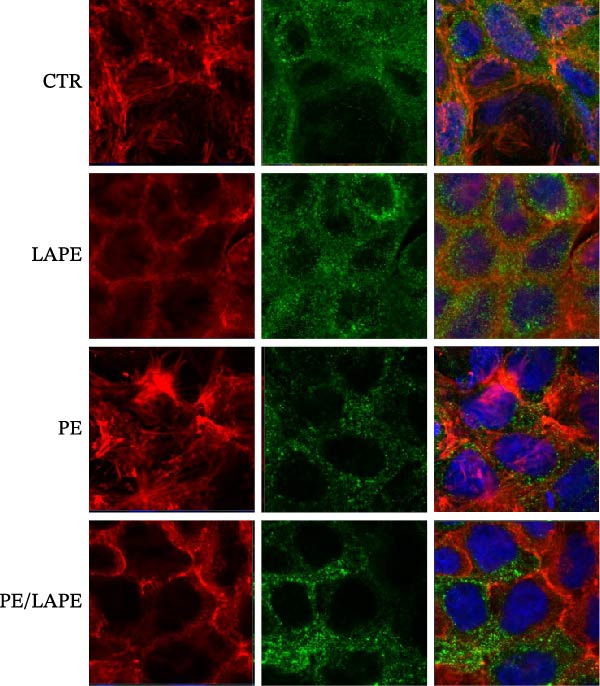
Confocal analysis of F‐actin (red) and β‐catenin (green) expression in D‐Caco‐2 cells. after 72 h incubation without (CTR) and with PE, LAPE, and PE/LAPE combination. Cell nuclei were stained with DAPI. Images are representative of three independent experiments.

Lipid peroxidation can lead to alterations in the permeability of biological membranes, resulting in the disruption of normal cell functions [13]. This finding prompted us to investigate whether the increased lipid peroxidation induced by PE influences the EMT and dedifferentiation of D‐Caco‐2 cells. Specifically, we examined the role of PE in the cellular localization of F‐actin and alkaline phosphatase proteins in D‐Caco‐2 cells. After 72 h of PE‐treated D‐Caco2 cells, we observed the aggregation of F‐actin fibers in the cytoplasm compared to CTR cells (Figure 4). Additionally, a dedifferentiation process was evident in PE‐treated D‐Caco‐2 cells, as indicated by a decreased ALP protein signal detected by both confocal microscopy and ALP activity compared to CTR (Figure 5 panels A and B *p* = 0.0025 CTR vs. PE). Interestingly, the redistribution of F‐actin and the reduction of the ALP signal were partially reversed after 72 h of treatment with the PE/LAPE combination compared to PE‐treated D‐Caco‐2 cells.

Figure 5(a) Confocal analysis of ALP (green) expression in D‐Caco‐2 cells after 72 h incubation without (CTR) and with PE, LAPE, and PE/LAPE combination. Cell nuclei were stained with DAPI. Images are representative of three independent experiments. (b) ALP activity expression in D‐Caco‐2 cells after 72 h incubation without (CTR) and with PE, LAPE, and PE/LAPE combination.  ^∗∗^ *p* < 0.01, vs. PE alone.

**Figure:**
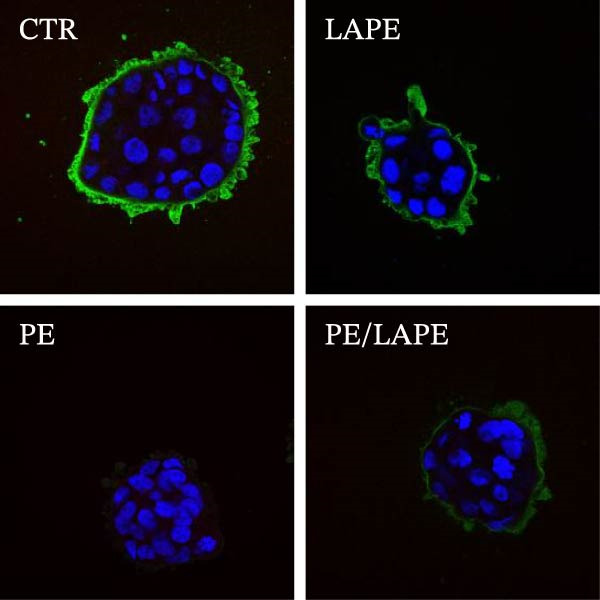
(a)

**Figure:**
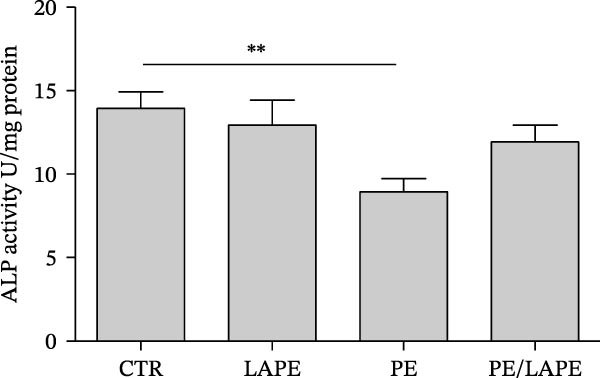
(b)

## 4. Discussion

MPs, through ingestion of sea and terrestrial food, come into contact with the human GI barrier [14], and they can have some negative effects on the human gut [10, 15–17]. Few studies have been performed utilizing EMPs representative of the ingested form. The purpose of this study was to evaluate, in vitro, the effects of EPE on differentiated human colorectal adenocarcinoma cell lines (D‐Caco‐2), which are like enterocytes and can mimic the small intestine of the GI system. The commune mechanisms of MPs toxicity include cell membrane disruption, their internalization, and ROS production [18]. In this study, we report the adverse effects of EMPs‐PE on viability, lipid peroxidation, permeability, and barrier function in D‐Caco‐2 cell monolayers.

Caco‐2 can take up particles between 0.5 and 10 µm by phagocytosis [19]. In our experimental condition, the EPE of 2.6 μm size affected D‐Caco‐2 cell viability in a dose‐ and time‐dependent manner. The IC50 value was reached at 2.5 μg/mL after 72 h of incubation. Interestingly, when PE (5 μg/mL) concentrations increased, the D‐Caco‐2 cell viability returned to the same value as the D‐Caco‐2 CTR. This effect may be due to the aggregation capacity of PE in an aqueous solution, which could contribute to decreased cellular uptake [20]. In animal studies, exposure to MPs has been shown to cause intestinal barrier dysfunction through ROS production [21]. Dietary polyphenols are emerging as prophylactic and therapeutic agents against disorders involving free radicals at the intestinal level; these compounds may help to protect the GI tract against damage by ROS present in foods or generated during food digestion. In our previous study [9], we demonstrated the GI protective role of a polyphenol‐rich apple extract from *Malus domestica* cv. Limoncella (LAPE) in a dinitro‐benzenesulfonic acid (DNBS)‐induced colitis in mice. LAPE improved the serum lipid peroxidation and reduced the NF‐κB activation. These results suggest that LAPE, through its antioxidant and anti‐inflammatory properties, could prevent damage induced by inflammatory bowel disease. Polyphenol‐rich extracts, including LAPE, may display natural variability depending on cultivar, harvest conditions, and extraction parameters. The LAPE extract used in the present study was previously fully characterized and described in detail in our earlier publication Lama et al. [9]. Polyphenols are known to undergo extensive GI transformation, which strongly influences their bioavailability and biological activity. In this study, it is important to note that LAPE was subjected to in vitro simulated GI digestion prior to its experimental use. This process reproduces the physiological conditions of the oral, gastric, and intestinal phases, generating metabolites that more closely reflect the compounds reaching the intestinal mucosa in vivo. GI digestion induces structural modifications of native polyphenols, including hydrolysis and oxidation, resulting in smaller, more absorbable molecules that retain or even enhance anti‐inflammatory and antioxidant activity. Therefore, the biological effects observed in our in vitro cellular model are likely mediated not only by catechin, epicatechin, and chlorogenic acid, which were present also after GI digestion, but also by their digestion‐derived metabolites, which have been widely reported to contribute to intestinal barrier protection and inflammatory modulation [11]. The utilization in this study of in vitro GI‐digested LAPE thus increases the physiological relevance of our findings.

So, as a further part of our study, we investigated the ability of LAPE to prevent the cytotoxic effects of PE in both D‐Caco‐2 and H_2_O_2_‐treated D‐Caco‐2 cells (DH‐Caco‐2). In both PE‐treated D‐Caco‐2 and DH‐Caco‐2 cells, cytotoxicity was due to an increase in lipid peroxidation, as evaluated by the TBARS assay. However, in LAPE alone and PE/LAPE combination‐treated DH‐Caco‐2 cells, the TBARS concentration significantly decreased compared to PE‐treated cells.

Several studies have indicated that natural antioxidants can also exhibit pro‐oxidant activity in the presence of metal ions [22]. Interestingly, when D‐Caco‐2 cells were incubated with LAPE alone, the TBARS concentration increased compared to CTR. This discrepancy in LAPE’s effects between D‐Caco‐2 and DH‐Caco‐2 cells may be due to our experimental cellular model. In fact, D‐Caco‐2 cells express digestive enzymes and transporters in the brush‐border membrane, which is characteristic of small intestinal absorptive epithelial cells. These polarized cells contain transporters for endogenous and apically accumulated nutrients, as well as other dietary compounds, along with receptors for many hormones, growth factors, and metal transport proteins such as Divalent Metal Transporter 1 (DMT1) [23]. However, it is important to note that the increase in TBARS concentration in LAPE‐treated D‐Caco2 cells did not lead to a decrease in cell viability.

When H_2_O_2_ induces oxidative damage in Caco‐2 cells, the permeability of the cell membrane increases, promoting the release of intracellular LDH into the external environment [23]. Therefore, we used LDH activity to reflect the degree of oxidative damage in DH‐Caco‐2 cells. In PE‐treated DH‐Caco‐2 cells, intracellular LDH release significantly increases. However, treatment with either LAPE alone or the PE/LAPE combination completely reversed LDH release. This indicates that LAPE, thanks to its antioxidant capacity, is effective in counteracting the cytotoxicity of PE induced by oxidative stress evaluated by lipid peroxidation. Recent papers have demonstrated that PE MPs can trigger inflammation after damaging membranes by increasing oxidative stress [24]. The intact intestinal barrier is important to prevent cell damage and restore cellular function caused by oxidative stress [25]. The permeability of the barrier after the 72 h treatment was investigated by measuring the lactulose/mannitol ratio on the basolateral side. The PE treatment increased permeability, resulting in a dysfunctional barrier in D‐Caco‐2 cells. However, permeability was significantly improved by PE/LAPE treatment, indicating the restoration of the barrier function in D‐Caco‐2 cells.

The increase in lipid peroxidation induced epithelial cell damage, leading to a breakdown of cellular tight junctions, reorganization of the actin cytoskeleton, dedifferentiation, and, in turn, inflammation. Previous studies have demonstrated that treatment with other MPs can improve cancer disease progression [26].

The morphology of the cytoskeleton was visualized by F‐actin and β‐catenin immunofluorescent staining, while tight junctions were evaluated through occludin expression [27–29] by western blot analysis. PE treatment disorganized the F‐actin fibrils and decreased occludin expression in D‐Caco2 cells, whereas the addition of LAPE clearly restored both F‐actin organization and occludin expression in the D‐Caco‐2 cells. The F‐actin disorganization, together with the decrease in tight‐junction associations induced by PE, may confer malignant properties to these cells. Clinical and experimental experiences indicate that differentiation and malignancy are inversely correlated. Here, we evaluated the differentiation of D‐Caco‐2 cells by confocal microscopy and activities of brush‐border enzyme ALP. In the D‐Caco‐2 cells cultured in the presence of PE, both ALP organization and activity decreased; this effect was reverted by LAPE treatment. The present findings are consistent with a mechanistic model in which PE‐induced oxidative stress initiates lipid peroxidation, leading to membrane instability and activation of signaling pathways that promote EMT. Increased peroxidation products such as MDA are known to activate NF‐κB, MAPKs (ERK1/2, JNK, and p38), and TGF‐β/SMAD signaling, all of which drive cytoskeletal remodeling, tight‐junction disassembly, and loss of epithelial polarity. The disruption of F‐actin and reduction of occludin expression observed in PE‐treated cells aligns with this mechanism. The protective effects of LAPE may rely on its ability to scavenge ROS, suppress NF‐κB activation, and stabilize membrane lipids, thereby preventing activation of these EMT‐related pathways.

A limitation of this study is the exclusive use of differentiated Caco‐2 cells, which, although widely adopted as an intestinal epithelial model, originate from a colorectal adenocarcinoma. Their cancer‐derived phenotype may affect basal oxidative status, metabolic pathways, and responsiveness to xenobiotic stressors. Therefore, while the effects observed here reflect canonical epithelial responses to MP‐induced oxidative stress, future studies should include nontransformed intestinal models, human primary epithelial cultures, or intestinal organoids to confirm the translational relevance of our findings.

## 5. Conclusions

The results of this study highlight the cytotoxic effects of PE on D‐Caco‐2 cells, leading to a dose‐ and time‐dependent reduction in cell viability and an increase in lipid peroxidation. Intestinal barrier disruption was confirmed by increased permeability and the disorganization of tight junctions, suggesting a potential contribution of MPs to intestinal dysfunction and inflammation. However, treatment with LAPE exhibited a protective effect, reducing oxidative damage and restoring intestinal barrier functionality. These findings suggest that polyphenols could be a promising option to mitigate the negative effects of MPs on the intestine, paving the way for future research into their potential therapeutic applications.

## Author Contributions

Conceptualization: Paola Stiuso, Stefania Lama, Massimo Venditti, Gian Carlo Tenore, and Mohamed Banni. Methodology: Alessandra Biasi, Carmen Lenti, and Hana Najahi. Validation: Stefania Lama, Carmen Lenti, and Alessandra Biasi. Formal analysis: Stefania Lama, Carmen Lenti, and Alessandra Biasi. Investigation: Stefania Lama, Carmen Lenti, and Alessandra Biasi. Writing – original draft preparation: Paola Stiuso, Stefania Lama, Massimo Venditti, and Gian Carlo Tenore. Writing – review and editing: Paola Stiuso, Stefania Lama, and Massimo Venditti. Supervision: Ettore Novellino. Funding acquisition: Paola Stiuso.

## Funding

This research was funded by the Department of Precision Medicine PATG.Assegnazioni 2024. Open access publishing facilitated by Universita degli Studi della Campania Luigi Vanvitelli, as part of the Wiley ‐ CRUI‐CARE agreement.

## Disclosure

All authors have read and agreed to the published version of the manuscript.

## Conflicts of Interest

The authors declare no conflicts of interest.

## Data Availability

The data that support the findings of this study are available from the corresponding author upon reasonable request.
